# Differential effects of diazepam and MPEP on habituation and neuro-behavioural processes in inbred mice

**DOI:** 10.1186/1744-9081-8-30

**Published:** 2012-06-11

**Authors:** Amber R Salomons, Nathaly Espitia Pinzon, Hetty Boleij, Susanne Kirchhoff, Saskia S Arndt, Rebecca E Nordquist, Lothar Lindemann, Georg Jaeschke, Will Spooren, Frauke Ohl

**Affiliations:** 1 Department of Animals in Science and Society, Division of Animal Welfare and Laboratory Animal Science, Faculty of Veterinary Medicine, Utrecht University, Yalelaan 2, 3584, Utrecht, CM, The Netherlands; 2 Rudolf Magnus Institute of Neuroscience, Universiteitsweg 100, Utrecht, CG, 3584, The Netherlands; 3 Department of Farm Animal Health, Faculty of Veterinary Medicine, Utrecht University, Yalelaan 7, Utrecht, CL, 3584, The Netherlands; 4 F. Hoffmann-La Roche Ltd. Pharmaceuticals Division, Discovery Neuroscience, Basel, Switzerland

**Keywords:** Anxiety, Behavior, Benzodiazepines, C-Fos, Cortisosterone, mGluR5 antagonist

## Abstract

**Background:**

Previous studies have demonstrated a profound lack of habituation in 129P3 mice compared to the habituating, but initially more anxious, BALB/c mice. The present study investigated whether this non-adaptive phenotype of 129P3 mice is primarily based on anxiety-related characteristics.

**Methods:**

To test this hypothesis and extend our knowledge on the behavioural profile of 129P3 mice, the effects of the anxiolyticdiazepam (1, 3 and 5 mg/kg) and the putative anxiolytic metabotropic glutamate receptor 5 (mGlu5R) antagonist 2-methyl-6-(phenylethynyl)pyridine (MPEP, 3, 10 and 30 mg/kg) treatment on within-trial (intrasession) habituation, object recognition (diazepam: 1 mg/kg; MPEP 10 mg/kg) and on the central-nervous expression of the immediate early gene c-Fos (diazepam: 1 mg/kg; MPEP 10 mg/kg) were investigated.

**Results:**

Behavioural findings validated the initially high, but habituating phenotype of BALB/c mice, while 129P3 mice were characterized by impaired intrasession habituation. Diazepam had an anxiolytic effect in BALB/c mice, while in higher doses caused behavioural inactivity in 129P3 mice. MPEP revealed almost no anxiolytic effects on behaviour in both strains, but reduced stress-induced corticosterone responses only in 129P3 mice. These results were complemented by reduced expression of c-Fos after MPEP treatment in brain areas related to emotional processes, and increased c-Fos expression in higher integrating brain areas such as the prelimbic cortex compared to vehicle-treated 129P3 mice.

**Conclusions:**

These results suggest that the strain differences observed in (non)adaptive anxiety behaviour are at least in part mediated by differences in gamma-aminobutyric acid- A and mGluR5 mediated transmission.

## Background

Adaptive anxiety in mice may be characterised by changes in behavioural responses over time, for example habituation to a novel environment. In contrast, non-adaptive anxiety might be mirrored by a lack of such a habituation, a phenomenon which may severely interfere with the normal interaction of the animal with its physical and social environment [1-3].

Recently, we found that 129P3/J mice are characterized by a profound lack of habituation to the modified hole board test as shown by highly increased avoidance behaviour over time while BALB/c mice, which have been reported to be highly anxious [4,5], show rapid habituation to the same test environment [2]. In addition, in 129P3/J mice c-Fos expression was found to be lower after the habituation procedure in distinct brain areas (e.g. prelimbic cortex and lateral septum) in comparison to BALB/c mice [2]. In a subsequent study we further demonstrated that exposure to chronic mild stress prior to repeated behavioural testing intensified the lack of habituation also in other behavioural parameters such as locomotion [6]. Other studies have found specific behavioural characteristics in 129P3 mice, such as less locomotor activity and more anxiety-related behaviour compared to for example C57BL/6 mice [7,8]. From these and our results we concluded that 129P3/J mice may represent an interesting animal model for non-adaptive anxiety.

However, it remains unclear whether the profound lack of habituation in 129P3/J mice is primarily based on anxiety-related characteristics. For example, avoidance behaviour can be confounded by other motivational systems, such as exploration [7,9,10] or cognitive processes [3,11,12]. We hypothesise that if the habituation profile in 129P3 mice was primarily based on anxiety-related characteristics, anxiolytic treatment should improve the habituation capacity in these mice.

To test this hypothesis, two anxiolytic compounds were used in the present study: the benzodiazepine Diazepam as a standard anxiolytic, and the metabotropic glutamate receptor 5 antagonist (mGlu5R) MPEP (2-methyl-6-(phenylethynyl)pyridine). Diazepam has shown anxiolytic efficiency over decades [13,14]. However, this compound is known to induce side effects at the cognitive level i.e. amnesia as well as in activity sedation [13-15]. Therefore, the putative anxiolytic MPEP was included in the present study. A number of studies have shown anxiolytic properties of MPEP [16-18]. For example, MPEP significantly reduced fear potentiated startle [18] and increased the number of open arm entries in the elevated plus maze, similar as treatment with diazepam [19] Furthermore, treatment with MPEP attenuated stress-induced hyperthermia and decreased the number of buried marbles in a marble burying test, whereas no effect of MPEP treatment was observed on spontaneous locomotor activity [18].

Since our previous studies evaluated between trial or intersession habituation, we were now interested in extending our knowledge about the behavioural profile in 129P3/J mice by investigating within-trial intrasession habituation as well. It has been suggested that intersession habituation reflects memory or retention of the previous exposures, while intrasession habituation might indicate adaptive capacity [12].

In the present study, the two mouse strains BALB/c and 129P3/J, were tested after acute treatment with either diazepam or MPEP for 30 minutes in the open field (intra-session habituation), and an object recognition test (cognitive performance). In addition to behavioural parameters, levels of plasma corticosterone (CORT) were determined before and after behavioural testing. Finally, the expression of c-Fos, a marker for neural activity [20,21], was investigated after behavioural testing in brain areas involved in emotional and cognitive processing. Based on the hypothesis that the non-adaptive phenotype of 129P3 mice is primarily anxiety-related, we expected anxiolytic treatment to facilitate habituation and decrease post-testing corticosterone levels. In addition we expected enhanced c-Fos expression in brain areas involved in the integration of emotional and cognitive processes.

## Methods

### Animals and housing

Naive male BALB/cJ (BALB/c, stock nr. 000651) and 129P3/J (129P3, stock nr. 000690) were obtained from the Jackson Laboratory (Bar Harbour, Maine, USA) and housed individually in Eurostandard Type II cages (size: 26.7 × 20.7 × 14 cm, Tecniplast, Buguggiate, Italy) provided with bedding material (Lignocel®, J. Rettenmaier & Söhne GmbH, Germany), a tissue (KLEENEX® Facial Tissue, Kimberly-Clark Professional BV, Ede, The Netherlands) and a shelter for cage enrichment. Mouse chow (CRM, Expanded, Special Diets Services Witham, England) and tap water were available *ad libitum*. For all experiments, the mice were acclimated to the experimental room for 17 days at the animal facilities of The Netherlands Vaccine Institute (Bilthoven, The Netherlands) under a reversed dark/light cycle (lights on between 18.00 h and 6.00 h). A radio played constantly as background noise (radio music interspersed with talk-shows, +/−60 dB). During this period the animals were handled three times a week by the person who also performed the behavioural tests. All behavioural testing took place between 9.00 and 13.00 h in the animal’s housing room and equipment (including the behavioural test set-ups) was installed before the animals arrived. Relative humidity was kept at a constant level of approximately 50%, room temperature was sustained at 22 °C ± 2 and ventilation rate was 15–20 air changes per hour.

The experimental protocols were approved by the Animal Experiments Committee of the Academic Biomedical Centre Utrecht, The Netherlands. Furthermore, all animal experiments followed the ‘Principles of Laboratory Animal Care’ and refer to the Guidelines for the Care and Use of Mammals in Neuroscience and Behavioural Research (National Research Council 2003).

### Drugs

Diazepam (BUFA, The Netherlands) and 2-methyl-6-(phenylethynyl)-pyridine (MPEP, Hoffmann-La Roche, Basel, Switzerland) were prepared in 0.1 % Tween 80 and saline in a volume of 10 ml/kg and injected i.p. in the experimental room 30 minutes prior to behavioural testing. Vehicle treatment consisted of 0.1 % Tween 80 and saline and was injected i.p. 30 minutes prior to behavioural testing. Anxiolytic effects after diazepam treatment in BALB/c mice have been found in a dose range of 1–5 mg/kg [22]. For MPEP treatment, anxiolytic effects have been found in a dose range of 3–30 mg/kg [17,18]. There is no knowledge about diazepam and MPEP effects in 129P3 mice specifically so a dose response curve was included in the open field test.

### Experiment 1: the open field (OF)

A total of 56 mice per strain were used and randomly assigned to 3 different dose groups per compound and one vehicle group (n = 8 per treatment group). Animals were tested with either diazepam (1, 3 or 5 mg/kg), MPEP (3, 10 or 30 mg/kg) or vehicle. While we used one vehicle group only in order to decrease the number of animals needed, the diazepam or MPEP treated mice were considered as parallel experiments and were thus analyzed per compound. Although, all treated mice were tested conjointly in a given test session in order to prevent variability of baseline behaviours among experiments. For all animals, diazepam MPEP or vehicle was i.p. injected 30 minutes before behavioural testing. Thirty minutes after behavioural testing a blood sample was taken and 120 minutes after testing the animals were euthanized by decapitation and brains were removed.

#### Apparatus

The OF apparatus consisted of a circular grey PVC arena, 80 cm in diameter and 33 cm high grey PVC walls (Additional file 1: Figure A1a). The arena was divided by red concentric circles in an outer zone, inner zone and centre area. Extra lines radiating out from the centre were placed on the floor as indicator for locomotion. One extra light bulb (red light) was fastened above the arena making the light intensity in the OF about 5–10 lux. Each animal was individually placed in the OF for 30 minutes, always starting from the same position at the edge of the OF. Behaviour was directly monitored and scored by a trained observer blind to treatment group using the program Observer 5.0 (Noldus Technology, The Netherlands). After each trial the OF was carefully cleaned with tap water and a towel. Behaviours scored in the OF included avoidance behaviour of the centre area, risk assessment, locomotor and exploratory behaviour and arousal. A description of all behaviours measured can be found in Table 1.

**Table 1:** Overview of behavioural parameters measured in the open field and the object recognition test

	**Behavioural parameter**	**Abbreviation**	**Behavioural dimension**
Experiment 1: Open Field	Latency until the first centre entry	Latency centre	Avoidance behaviour
	Total time spent in centre	Centre duration	
	Total number of centre entries	Centre entries	
	Total number of stretched attends	Stretched attends	Risk assessment
	Latency until first stretched attend	Latency stretched attend	
	Total number of line crossings	Line crossings	Locomotion
	Latency until first line crossing	Latency line cross	
	Total time spent immobile	Immobility duration	
	Latency until the first immobility event	Latency immobility	
	Total number of rearings	Rearings	General exploration
	Latency until first rearing	Latency rear	
	Total time spent grooming	Grooming duration	Arousal/de-arousal
	Latency until the first grooming event	Latency grooming	
	Total number of fecal boli	defecations	
Experiment 2: Object recognition test	Discrimination index*	DI	Object memory
	Latency until first exploration novel object	Latency novel object	
	Latency until first exploration familiar object	Latency familiar object	
	Total time spent exploring novel object	Time novel object	
	Total time spent exploring familiar object	Time familiar object	
	Total number of stretched attends	Stretched attends	Risk assessment
	Total number of line crossings	Line crossings	Locomotion
	Total time spent immobile	Immobility duration	
	Latency until the first immobility event	Latency immobility	
	Total number of rearings	Rearings	General exploration
	Total time spent grooming	Grooming duration	Arousal/de-arousal
	Latency until the first grooming event	Latency grooming	

* The DI was calculated as followed: (total exploration time novel object – total exploration time familiar object) / total time spent exploring novel + familiar object.

#### Blood samples

Basal blood samples (*basal)* were collected 4 days before the start of the OF using tail vein incision for corticosterone (CORT) determination. Thirty minutes after behavioural testing a second blood sample was taken (*non-basal*). For blood sampling, mice were transported individually in their home cage to an adjacent laboratory (in order not to disturb circadian rhythm of the mice, the hallway and rooms were under red light conditions). By using tail vein incision a small blood sample was collected (±50 μl) and stored in prechilled Microvette tubes (CB300, Sarstedt, Numbrecht, Germany) containing lithium heparin. Blood samples were centrifuged (10 min at 12000 rpm, 4 °C) and stored at −20 °C until measurement. CORT levels were measured by radioimmunoassay (RIA) according to the protocol of the supplier with an ImmuChem™ Double Antibody Corticosterone kit for rats and mice (MPI Biochemicals, Amsterdam, The Netherlands).

#### c-Fos

Brains from treatment groups: vehicle, 1 mg/kg diazepam and 10 mg/kg MPEP were removed for c-Fos immunohistochemistry. After removal, brains were frozen in liquid (−80 °C) 2-methylbutane which was cooled with dry ice and stored at −80 °C. Coronal sections were cut (20 μm) and mounted on Menzel SuperFrost Plus slides (Menzel GmbH & Co, Braunschweig, Germany) and stored at −20 °C. The sections were processed for c-Fos immunohistochemistry as described previously [2], dilution with a polyclonal primary antibody (1:1000, SC-52 Santa Cruz Biotechnology, Santa Cruz, USA), and a donkey-anti-rabbit IgG Biotin SP conjugated secondary antibody (1:400, Jackson ImmunoResearch Laboratories, Inc USA). Cells containing a nuclear brown-black reaction product were considered as c-Fos positive cells and counted in several brain areas which are known to be involved in anxiety [23-25]: medial prefrontal cortex (prelimbic, *PrL*), lateral septum (dorsal, *LSD*; intermediary, *LSI*; ventral, *LSV*), bed nucleus of the stria terminalis (medial anterior, *BSTMA*; lateral posterior, *BSTLP*; medial ventral, *BSTMV*), hippocampus (granular layer dentate gyrus, *DG*), hypothalamus (paraventricular nucleus, *PVN;* dorsal medial hypothalamus, *DMH*), amygdala (basolateral nucleus, *BLA*; central nucleus *cAmy*) and the periaqueductal gray (dorsolateral, *dlPAG*; dorsomedial, *dmPAG*; lateral, *lPAG*; ventrolateral, *vlPAG*). The anatomical localization was aided by use of adjacent Nissl stained sections and the illustrations in a stereotaxic atlas [26]. For each region at least two overt landmarks were used. For quantitative analysis of c-Fos positive cells, the program Leica Qwin (image processing and analysis software, Cambridge, United Kingdom) was used. Left and right hemispheres were analyzed for stained neurons per mm^2^ and calculated for one section separately and averaged for each animal.

### Experiment 2: the object recognition test (ORT)

Results from the open field led to the conclusion that 1 mg/kg diazepam and 10 mg/kg MPEP could be used as the minimal effective dose range, without causing side effects in both strains. We used these doses to investigate object memory. A total of 27 mice per strain was used for the ORT (n = 9 per treatment group). On day 1, the familiar object was placed in the home cage. On day 2, the animals were i.p. injected with diazepam (1 mg/kg), MPEP (10 mg/kg) or vehicle 30 minutes before behavioural testing. Thirty minutes after the ORT, a blood sample was taken and 120 minutes after testing the animals were euthanized by decapitation.

#### Apparatus

The test apparatus was a Eurostandard Type II L cage (size: 36.5 x 21 x 14 cm; Tecniplast, Buguggiate, Italy) without any bedding and equally divided in 6 squares by black lines on the floor (Additional file 1: Figure A1b). Light conditions were the same as in the experimental room, red light with an illumination intensity of approximately 5 lux. Two different objects were used that differed in colour, material and shape (screw nut and die). Both objects were considered too heavy to be displaced by the animals. All animals were allowed to familiarize with one of the two objects for 24 h in their home cage (randomized for each strain and treatment i.e. half the animals of each testing group received a nut, the other half received a die) one day before the ORT. Twenty-four hours later, the animals were tested in a one-trial ORT, without habituation to the test arena. This was done to study the effect of anxiolytic treatment on object memory *per se*. Both objects (for the familiar object a duplicate was used) were always placed in the same corner of the apparatus, each was positioned at the same distance from the wall. For testing, the animals were individually placed in the apparatus always in the same corner opposite of the objects and scored by a trained observer blind to treatment group for 10 minutes. After each trial the ORT was carefully cleaned with tap water and a towel. Behaviours scored in the ORT included object memory, risk assessment, locomotor and exploratory behaviour and arousal. A description of all behaviours measured can be found in Table 1.

#### Blood samples

Basal blood samples (*basal)* were collected 4 days before the start of the ORT using tail vein incision for corticosterone (CORT) determination. Thirty minutes after the ORT a second blood sample was taken (non-basal). The same procedure and analyses were used as described in the OF (section 2.3.2).

### Statistics

All statistical analyses were carried out according to Field [27] using the software program SPSS® for Windows (version 16.0.1; SPSS Inc., IL, USA). Two-sided, exact i.e. for the non-parametric tests probabilities were estimated throughout. Continuous numerical data (CORT, c-Fos, latency and relative duration of behavioural parameters) were summarized as means with standard error of the mean (SEM), whereas discrete data on the ordinal scale (total number of behavioural parameters) were represented as medians with the interquartile range (IQR). The Kolmogorov-Smirnov one-sample test was used to check Gaussanity of the continuous numerical data. Several parameters that were not normally distributed were transformed to a Gaussian distribution by using a mathematical function or by rank transformation [28]. Discrete numerical data (total numbers of the behavioural parameters of the open field) were fist rank-transformed. Behavioural numerical data from the open field and CORT values were tested for significant differences by multivariate repeated measures ANOVA [29]. Tests of significance were derived using the Wilk’s lambda criterion (for the open field, time interval was taken as within-subject factor and strain and dose as between-subject factors). For CORT values the basal/non-basal CORT was taken as within-subject factor and strain and dose as between-subject factors. For latency and defecation data of the OF, ORT data and c-Fos results, a two-way ANOVA was used with strain and treatment (or dose for the open field) as main between-subject factors. If ANOVA detected significant effects, group means were further compared. Between-subject *post hoc* comparisons were done with either unpaired Student’s t tests for normally distributed data, or for non-normally distributed data and for discrete data, the same comparisons were performed using a Wilcoxon-Mann–Whitney test. For the ORT this was done to investigate whether the discrimination index differed significantly from zero i.e. no discrimination between novel and familiar object. To take the greater probability of a Type I error due to multiple hypotheses into account, we calculated for each behavioural category separate so-called Dunn-Šidák corrections: ANOVA’s: α = 1 – [1–0.05]^1/q^; q = number of parameters per behavioural category; *post hoc* Student’s t tests, the Wilcoxon-Mann–Whitney tests, and Wilcoxon matched-pairs signed ranks tests: α = 1 – [1 – 0.05]^1/q^; q = number of parameters per behavioural dimension multiplied by the number of times a group is used for a meaningful comparison. The corrected thresholds used for the ANOVA’s and *post hoc* comparisons can be found in the 1: Table A1 (open field) and Additional file 1: Table A2 (object recognition test).

## Results

### Open field

#### Behaviour

In general, vehicle-treated BALB/c mice initially showed more avoidance behaviour of the unprotected centre of the OF compared to vehicle-treated 129P3 mice. While BALB/c mice showed a decrease in avoidance behaviour across the experimental period, 129P3 mice did not, confirming a lack of habituation in these mice. Whereas diazepam treatment decreased initial anxiety in BALB/c mice, higher doses caused clear immobility in 129P3 mice. In contrast, MPEP exerted only minor behavioural effects in both strains.

##### Diazepam

**Figure 1:**
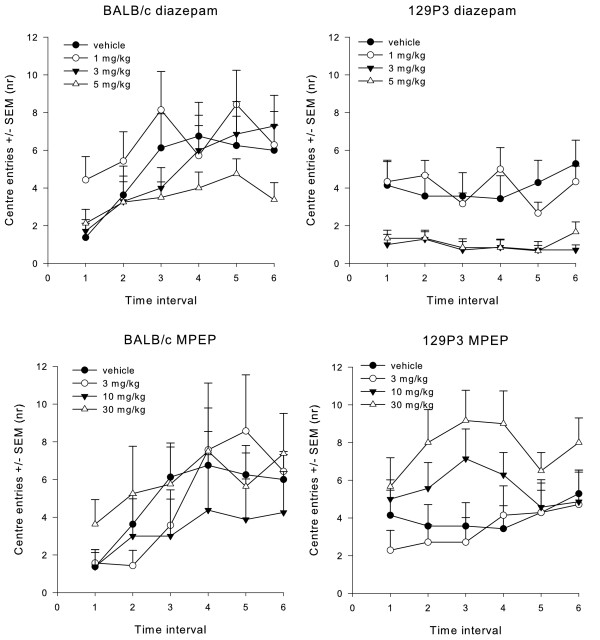
**The number of centre entries during open field testing.** Animals were pre-treated with vehicle, diazepam or MPEP. Data are displayed as the mean number (± SEM) of centre entries during each time interval of 5 min each. Significant time effects were found after both treatments.

*Avoidance behaviour*: treatment with diazepam did not affect the latency until the first centre entry. In contrast, a time interval effect (F_5;48)_ = 2.559, P < 0.05) was found for the time spent in the centre. Furthermore, a time interval (F_5;52)_ = 3.817, P < 0.05) and time x strain interaction (F_5;52)_ = 5.226, P < 0.0170) effect for the number of centre entries (Figure 1). Neither strain nor dose effects were found, suggesting that the time interval effect of the ANOVA was independent of these factors. Post hoc testing revealed no differences between trials in the time spent in the centre. Vehicle-treated BALB/c mice made more centre entries in time interval 6 when compared to time interval 1 (P < 0.01), thereby showing less avoidance behaviour across the experimental period. No *post hoc* effects were found in 129P3 mice, suggesting that neither vehicle-treated nor diazepam treated mice showed a change in avoidance behaviour across the experimental period.

**Figure 2:**
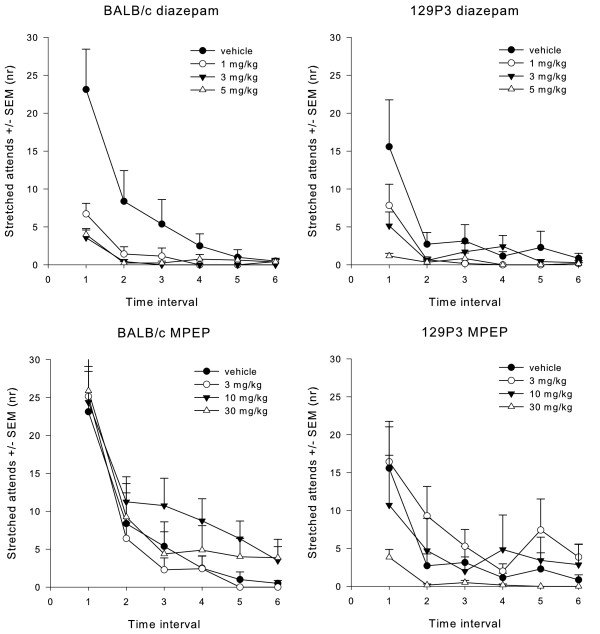
**The number of stretched attends during open field testing.** Animals were pre-treated with vehicle, diazepam or MPEP. Data are displayed as the mean number (± SEM) of centre entries during each time interval of 5 min each. Significant time and dose effects were found after diazepam treatment, but only time effects after MPEP treatments.

*Risk assessment:* ANOVA revealed a time interval effect (F_5;51)_ = 14.116, P < 0.05), dose effect (F_3;55)_ = 8.476, P < 0.05) and time interval x dose interaction (F_15;141.2)_ = 2.264, P < 0.05) effect for the number of stretched attends (Figure 2). *Post hoc* testing showed that 1, 3 and 5 mg/kg diazepam-treated BALB/c mice showed a significant decrease in the number of stretched attends across the time intervals (P < 0.01). Vehicle-treated animals of both strains showed a decrease in the number of stretched attend for the last time interval only (P < 0.01). These results suggest that diazepam especially decreased initial risk assessment behaviour, which was independent of strain. No effects were observed for the latency until the first stretched attend.

*Locomotor activity*: ANOVA revealed a significant strain (F_1;55)_ = 46.839, P < 0.05), and time interval x strain interaction (F_5;51)_ = 7.245, P < 0.05) effect for the number of line crossings. *Post hoc* testing showed an increase in the number of line crossings across the time intervals in vehicle-treated BALB/c mice (P < 0.01). No differences in the number of line crossings across the time intervals were observed in 129P3 mice. Whereas ANOVA detected no significant dose effects, BALB/c mice showed more line crossings than 129P3 mice after pre-treatment with 3 and 5 mg/kg (P < 0.01). No effects were found for the latency until the first line crossing. For immobility duration, a significant strain (F_1;55)_ = 17.084, P < 0.05), dose (F_3;55)_ = 8.076, P < 0.05) and strain x dose interaction (F_3;55)_ = 5.268, P < 0.05) effect were found. *Post hoc* testing revealed that 129P3 mice after treatment with 3 and 5 mg/kg diazepam spent more time immobile when compared to vehicle-treated mice (P < 0.01). No significant *post hoc* effects were observed in BALB/c mice, suggesting that only 129P3 mice were affected in their immobility duration after diazepam treatment. For the latency until the first immobility event, ANOVA revealed a significant dose (F_3;55)_ = 4.294, P < 0.05) effect. *Post hoc* testing showed that pre-treatment with 3 and 5 mg/kg diazepam decreased the latency until the first immobility event (P < 0.01).

Exploration and arousal behaviour effects can be found in the supplementary results. Also a general overview of all behavioural results from the OF after diazepam treatment can be found in Additional file 1: Table A3.

##### MPEP

*Avoidance behaviour*: ANOVA revealed strain (F_1;55)_ = 6.626, P < 0.05) differences in latency until the first centre entry. *Post hoc* testing revealed that BALB/c mice showed a higher latency to enter the centre area when compared to 129P3 mice (P < 0.01), suggesting more initial avoidance behaviour than 129P3 mice. For centre duration a time interval effect (F_5;51)_ = 5.332, P < 0.05) was observed. *Post hoc* testing showed increased time spent in the centre across the time intervals, although this was independent of strain or treatment. While ANOVA detected a time interval effect in the number of centre entries (F_5;51)_ = 6.334, P < 0.05, 1), *post hoc* testing did not reveal any significant differences between the time intervals.

*Risk assessment*: ANOVA detected a significant time interval effect (F_5;51)_ = 21.231, P < 0.05), and strain x time interval interaction (F_5;51)_ = 4.270, P < 0.05) effect for the number of stretched attends (Figure 2). *Post hoc* testing showed that the number of stretched attends decreased across the time intervals in both strains, although BALB/c showed more initial risk assessment behaviour when compared to 129P3 mice. Also, BALB/c mice showed more stretched attends after 10 mg/kg MPEP when compared to 129P3 mice (P < 0.05). No effects were observed for the latency until the first stretched attend.

*Locomotor activity:* after MPEP treatment, ANOVA revealed a significant time interval x strain interaction effect (F_5;51)_ = 6.651, P < 0.05). *Post hoc* testing showed a significant increase in the number of line crossings across the time intervals in BALB/c mice. No significant differences were observed in the number of line crossings between BALB/c and 129P3 mice. For latency until the first line crossing, a significant strain effect (F_1;55)_ = 10.418, P < 0.05) was observed, although *post hoc* testing did not reveal a significant difference between the strains. No significant effects were observed for immobility duration. For latency until the first immobility event, ANOVA detected a significant dose effect (F_3;55)_ = 5.314, P < 0.05). *Post hoc* testing showed that pre-treatment with 10 mg/kg significantly increased the latency until the first immobility event (P < 0.01), which was independent of strain.

Exploration and arousal behaviour effects can be found in the supplementary results. Also a general overview of all behavioural results from the OF after MPEP treatment can be found in Additional file 1 Table A4.

#### Corticosterone

**Figure 3:**
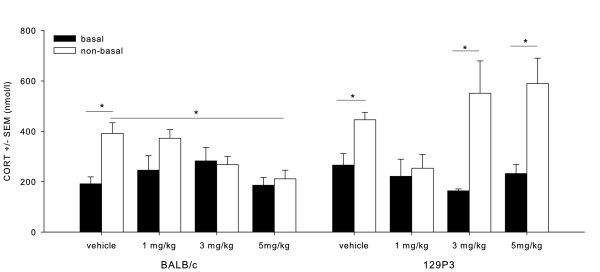
**Mean (± SEM) CORT levels after diazepam treatment.** CORT data are displayed before (basal) and after (non-basal) behavioural testing in the OF for BALB/c mice (left) and 129P3 mice (right). * = significantly different after *post hoc* comparisons.

Diazepam: basal/non-basal (F_1;53)_ = 33.331, P < 0.05), strain (F_1;53)_ = 5.285 , P < 0.05), dose (F_3;53)_ = 4.885 , P < 0.05), and strain x dose interaction (F_3;53)_ = 3.467, P < 0.05) effects were found (Figure 3). *Post hoc* testing showed that behavioural testing increased CORT levels, which was indicated by a significantly higher value for both BALB/c and 129P3 mice in vehicle-treated animals after testing (P < 0.01). In BALB/c mice, non-basal CORT levels decreased after 5 mg/kg diazepam as compared to non-basal levels in vehicle-treated BALB/c mice. Furthermore, 1 mg/kg diazepam decreased non-basal CORT values as compared to non-basal values of vehicle-treated 129P3 mice (P < 0.0052). The reverse was observed after pre-treatment with 3 and 5 mg/kg diazepam, which significantly increased CORT values compared to basal levels in 129P3 mice as shown by *post hoc* testing (P < 0.01, Figure 3).

**Figure 4:**
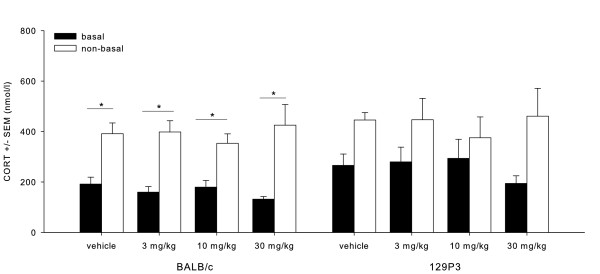
**Mean (± SEM) CORT levels after MPEP treatment.** CORT data are displayed before (basal) and after (non-basal) behavioural testing in the OF for BALB/c mice (left) and 129P3 mice (right). * = significantly different after *post hoc* comparison.

MPEP: basal/non-basal (F_1;47)_ = 29.661, P < 0.05) and strain effects (F_1;47)_ = 5.125, P < 0.05) were found (Figure 4). *Post hoc* testing revealed that CORT levels were generally higher after behavioural testing when compared to basal levels (P < 0.01). Additionally, *post hoc* testing revealed that 129P3 mice showed generally higher CORT levels as compared to BALB/c mice, although basal and non-basal values did not significantly differ in 129P3 mice, whereas BALB/c mice showed significantly higher non-basal CORT values (P < 0.01). No dose effects were found. See also Additional file 1: Table A5.

#### c-Fos immunohistochemistry

Brains from animals treated with vehicle, 1 mg/kg diazepam and 10 mg/kg MPEP were processed for c-Fos immunohistochemistry.

**Figure 5:**
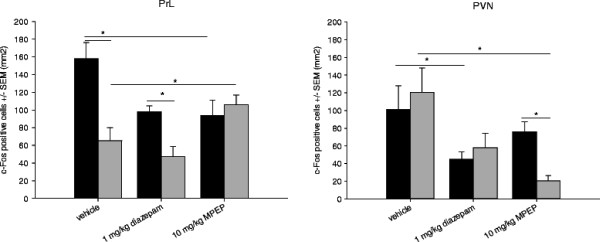
**Mean number of c-Fos positive cells (± SEM) after pre-treatment with vehicle, 1 mg/kg diazepam or 10 mg/kg MPEP treatment in the OF.** Data are displayed for the PrL (left) and the PVN (right). * = significantly different after *post hoc* comparison.

In vehicle-treated mice, strain differences were observed in the PrL (F_(1;79)_ = 18.377, P < 0.05, Figure 5a), LSD (F_(1;79)_ = 15.999, P < 0.01) and DG (F_(1;79)_ = 32.194, P < 0.05). BALB/c mice showed more c-Fos expression in these areas (P < 0.01) when compared to 129P3 mice, which is in accordance with our previous findings. After 1 mg/kg diazepam treatment, higher c-Fos expression was found in the PrL, DG and BLA in BALB/c mice compared to 129P3 mice (P < 0.01). Compared to vehicle-treated mice, diazepam-treated BALB/c showed less c-Fos positive cells in the PrL, PVN, LSD, and LSV (P < 0.01), while diazepam-treated 129P3 mice showed lower c-Fos expression in the PVN, BLA and DMH compared to vehicle-treated 129P3 mice. After MPEP treatment a different c-Fos expression pattern was observed. BALB/c mice showed more c-Fos positive cells in the PVN compared to 129P3 mice (P < 0.01, Figure 5b). Lower c-Fos expression was found after MPEP-treatment compared to vehicle treatment in BALB/c mice in the PrL, PVN, DMH, LSD, LSV, lPAG and vlPAG (P < 0.01). MPEP treatment in 129P3 mice induced lower c-Fos expression in the PVN, DMH, LSV, BLA and higher c-Fos expression in the PrL, DG, BSTMV and BSTMA compared to vehicle-treated 129P3 mice (P < 0.05). See also Additional file 1 Table A6.

### Object recognition test

#### Behaviour

**Figure 6:**
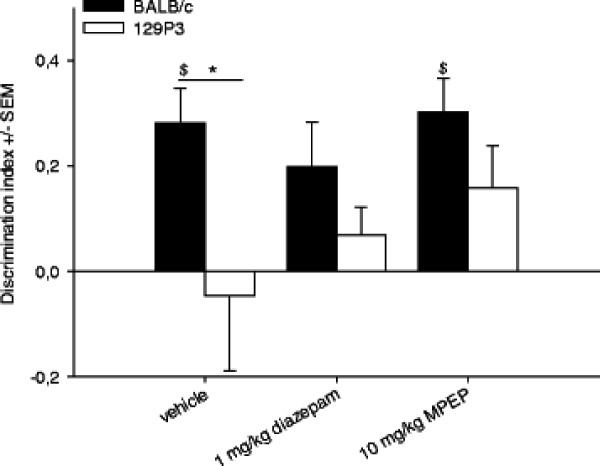
**Discrimination index (± SEM) after vehicle, diazepam or MPEP treatment in the ORT.** ANOVA revealed significant strain differences in DI (* = significantly different after *post hoc* comparisons); One-sample t-statistics revealed significant differences from zero ($ = significantly different from zero).

Strain differences were observed for the discrimination index (F_(1;44)_ =6.718, P < 0.05) and *post hoc* testing revealed a higher discrimination index in BALB/c mice compared to 129P3 mice after vehicle treatment (Figure 6). Additionally, within-subject *post hoc* comparisons were made to investigate whether the discrimination index was different from zero. This revealed that vehicle-treated BALB/c mice showed a positive discrimination index (t = 4.057, P = 0.01), indicating that these mice spend more time investigating the novel object compared to the familiar object. In contrast, vehicle-treated 129P3 mice showed no significant difference from zero, thereby demonstrating no difference between novel and familiar object exploration. Furthermore, *post hoc* testing showed that pre-treatment with diazepam, both strains showed no positive discrimination index, whereas MPEP did not affect discrimination between the objects in BALB/c mice. In contrast, pre-treatment with MPEP slightly increased the discrimination index in 129P3 mice, although this failed to reach significance (P < 0.054). No significant strain or treatment effects were found for the latency to explore the novel or the familiar object. Results from other behavioural parameters measured in the ORT can be found in Additional file 1 Table A7.

#### Corticosterone

Significant basal/non-basal (F_1;33)_ = 35.522, P < 0.05) and strain (F_1;33)_ = 23.344, P < 0.05) effects were found. Post hoc testing revealed that 129P3 mice had higher CORT levels than BALB/c mice. Furthermore, CORT levels were increased after behavioural ORT testing as compared to basal levels (P < 0.01). No significant treatment effects were found; see also Additional file 1: Table A8.

## Discussion

### General findings open field

Results from the present study demonstrated a significant intrasession habituation in BALB/c mice. In support of previous results [2] it seems that BALB/c mice indeed are characterized by an initially high but adaptive anxiety profile, which was shown by decreased anxiety-related behaviour over time. In contrast vehicle-treated 129P3 individuals failed to show intra-session habituation This was shown by a lack of change in avoidance, exploration and locomotor behaviour throughout the experimental period. Similar results were found by Tang et al. [8], who showed that BALB/c mice spent increased time in the centre of an open field during a 30 minute trial. The same study also demonstrated impaired habituation after 30 minutes open field testing in 129P3 mice. Valid evaluation of anxiety-related behaviour is suggested to be dependent on a comparable activity level [30].Both strains showed a comparable activity level at the onset of the testing trial, but only BALB/c mice increased their activity level over time. In contrast, such an increase was not observed in 129P3 mice. Apparently, the non-adaptive habituation phenotype in 129P3 mice determines their behaviour between as well as within sessions, a phenomenon found in different test systems [2,6,8], suggesting that this lack of habituation is a general characteristic in the 129P3 inbred strain, which is independent of their activity levels.

### Open field: effects of diazepam treatment

The general strain differences identified are further extended by the results of the pharmacological treatment. Whereas only minor treatment effects were observed on avoidance behaviour in the open field, the results nevertheless suggest a different response between the strains. Diazepam treatment decreased risk assessment behaviour and post-testing CORT levels only in BALB/c mice. Other studies have found anxiolytic effects of diazepam in BALB/c mice [22,31,32]. In contrast, diazepam treatment in 129P3 mice induced marked sedation. This difference in sensitivity towards diazepam treatment is likely to be due to strain characteristics in benzodiazepine receptor densities or sensitivity. BALB/c mice have been shown to exhibit a five-fold decrease in the density of benzodiazepine binding sites as compared to C57BL/6 mice [33]. While there are no reports on diazepam treatment in 129P3 mice, some pharmacological studies however have reported sedative effects of benzodiazepines in other 129 substrains. For example, Rodgers [30] found sedation in 129 S2 mice after chlordiazepoxide treatment and suggested that 129 S2 mice have an abnormal benzodiazepine/GABA-A receptor function. The same might be true for 129P3 mice as well. It is of note that the higher doses (3 and 5 mg/kg) of diazepam not only produced sedation in 129P3 animals but increased their post-testing CORT levels as well when compared to both vehicle-treated 129P3 mice and diazepam-treated BALB/c mice. This effect suggests either an activating effect on the HPA axis [34], or an emotionally aversive effect of the treatment-induced sedation in 129P3 animals [35]. Furthermore, acute diazepam treatment is known to cause hyperthermia in members of another substrain of the 129 family, 129 Sv mice [36]. Thus, diazepam might interfere with temperature regulation in 129P3 mice as well, which may be another explanation for the side effects observed. It should be mentioned though, that the diazepam doses used in the Open Field might have been too high. While a similar dose range has been used in BALB/c mice [4,22], it could very well be that sedation might be observed when animals are tested in their home cage. The apparent sedation in BALB/c mice could have been masked by increased vigilance in the behavioural test set-up.

### Open Field: effects of MPEP treatment

Notably, a different picture emerged after treatment with MPEP in the two strains. Avoidance, risk assessment and locomotor activity were unaffected by pre-treatment with MPEP in both strains. Lack of effect on locomotor behaviour corresponds to existing literature on MPEP [18,37]. Although anxiolytic effects of MPEP have been found in several animal models of anxiety [16-19], MPEP hardly had any effect on avoidance behaviour in both strains. Close inspection of MPEP treatment in 129P3 mice did reveal a decrease in avoidance behaviour, although it failed to reach significance. The anxiolytic effects of both compounds did show a difference between the strains; diazepam decreased risk assessment behaviour in both strains, whereas MPEP increased risk assessment behaviour in BALB/c mice, while it decreased in 129P3 mice. The relationship between risk assessment behaviour and avoidance behaviour was found to be independent as shown by factor analyses [38,39] Furthermore, it has been shown that risk assessment behaviour is very sensitive to anxiolytic drugs [40]. The existence of such a subtle anxiolytic effect of MPEP in 129P3 mice was further underlined by decreased post-testing CORT levels at all MPEP doses in 129P3 mice, an effect that could not be found in BALB/c mice.

### c-Fos

The strain-specific findings were extended by the results on c-Fos expression in different brain areas in the open field. Brain areas involved in the integration of emotional and cognitive processes, such as the PrL, DG, BST and LS [41-44] were found to be more active after MPEP treatment in 129P3 mice, whereas the Amy and PVN, brain areas primarily involved in emotional processes [45,46], showed lower c-Fos activity as compared to vehicle-treated 129P3 mice. In contrast, diazepam treatment in BALB/c mice especially resulted in decreased c-Fos expression in brain areas related to emotional processing, while no effects were found on higher cortical anxiety-related brain areas. Notably, strain differences in the PrL, DG and LSD in vehicle treated animals could not be found in MPEP treated animals.

An explanation for the strain-specific diazepam or MPEP treatment effects, respectively, is that diazepam and MPEP exert their anxiolytic effects via different mechanisms [47]. Given the fact that the two strains are further characterized by specific anxiety-phenotypes, distinct regulatory systems, underlying the specific phenotypes, may be suggested: diazepam is known to act inhibitory via the GABA-A system. In diazepam-treated BALB/c mice, we found c-Fos expression in the PVN, known to regulate HPA-axis activity, and the BLA, known to process anxiety-responses, to be reduced; these effects were not seen in 129P3 mice. In contrast, MPEP produced a different c-Fos expression profile in other brain areas in both strains, while 129P3 mice appeared to be more sensitive for MPEP.

mGlu5 receptors are highly expressed in several limbic structures such as the hippocampus, amygdala and septum [48,49] and activation of these receptors leads to excitatory effects in the brain, such as an increase of glutamatergic transmission [50-52]. A controlled glutamatergic transmission is known to be critical for higher order mental processes, whereas excessive glutamatergic transmission can lead to impairment of normal neural processes and even cell death [53,54]. Notably, it has been suggested that anxiety disorders may arise through excessive excitatory neurotransmission in response to stress [55]. For MPEP it has been shown that glutamate release is effectively reduced after acute treatment [56]. Interestingly, MPEP increased c-Fos expression in the PrL, and BST in 129P3 mice, when compared to vehicle treated animals, suggesting that higher order processes regulated by the prefrontal cortex are affected in addition to primarily emotional processes. From the present results as well as from our previous studies in 129P3 mice we conclude that the prelimbic cortex seems to play an important role in the phenotype of 129P3 mice. This is further supported by the notion that 129P3 mice display impaired fear extinction [57], a process which relies on a reciprocal connection between the prelimbic cortex and the amygdala [58]. The prelimbic cortex has been shown to act as the cognitive control system in emotional processing [41,59]. Given the fact that MPEP treatment increased c-Fos expression in the prelimbic cortex in 129P3 mice as compared to BALB/c mice and vehicle-treated 129P3 mice, it may be hypothesised that it is primarily the cognitive control over their emotionality which determines the behavioural profile of the 129P3 strain. However, at present we cannot exclude the possibility that the increased c-Fos expression is related to other neural processes and further studies are necessary to investigate which neurons become activated during the habituation procedure. In any case, it seems likely that reduced c-Fos expression in the prelimbic cortex in 129P3 mice is related to their non-adaptive anxiety phenotype.

### Object recognition

It has repeatedly been suggested that pathological, or non-adaptive anxiety may primarily be a specific, but not general, cognitive dysfunction [3,60]. To control for general cognitive functioning, we investigated the effects of the (putative) anxiolytic pre-treatment in a one trial object recognition test in both strains as well. As found earlier [2], BALB/c mice discriminated between the novel and familiar object. The fact that after diazepam treatment no positive discrimination index was found in this strain is likely to be explained by diazepam induced amnesic effects, which have been extensively reported [61,62]. In contrast to our earlier findings, vehicle-treated 129P3 mice showed no positive discrimination index, a discrepancy which may be explained by differences in experimental design: in our previous study, but not in the present one, animals were habituated to the test environment, which might be of impact on exploration behaviour especially in 129P3 mice. In this study we intentionally chose not to habituate the animals to the test environment, in order to investigate the effects of anxiolytic treatment on object memory *per se*. Although not statistically significant, the discrimination index indicates a trend towards a dose dependent improvement in object memory after MPEP treatment in 129P3 mice. This however would be in contrast to other findings that either no effects of MPEP are found on retrieval of object memory [37,63] or even impaired object recognition [64].

## Conclusions

Treatment with either diazepam or MPEP did not facilitate habituation in the open field and had only minor effects on anxiety-related behaviour in 129P3 mice. In contrast, the effect of MPEP on stress-induced CORT responses and c-Fos expression does support our hypothesis of a primarily anxiety-related phenotype in 129P3 mice. While these results seem contradictory at the first glance, several studies have found discrepancies between effects on physiological and behavioural readout-parameters of anxiety, with physiological measures sometimes being more sensitive [65]. It would thus be of interest to investigate anxiety-related parameters in 129P3 mice, which are not based on approach-avoidance behaviour [66]. In any case, based on the present findings it can be suggested that the observed strain differences in (non)adaptive anxiety behaviour are at least partly mediated by differences in GABAergic and mGluR5 mediated transmission. Further investigation of the functional role of these systems will help for a better understanding of the underlying neurobiological mechanisms of non-adaptive anxiety.

## Competing interests

The authors Dr. Will Spooren, Dr. George Jaescke and Dr. Lothar Lindemann are employees of F.Hoffmann-La Roche, a pharmaceutical company engaged in research and development of drugs for central nervous system disorders. The other authors declare no conflict of interest.

## Authors’ contributions

ARS, FO, REN and SSA designed the experiments and wrote the draft of the manuscript. ARS, NEP, HB and SK carried out the behavioural testing and performed analyses of the blood samples and c-Fos immunohistochemistry. LL, GJ, and WS provided MPEP and have given approval of the manuscript. All authors have read and approved the final manuscript.

## Supplementary Material

Additional file 1 Schematic representations of the open field and the object recognition test.Corrected P value thresholds of significance for the open field and object recognition test.Overview of all behavioural results in the open field after MPEP or diazepam treatment.Overview of CORT levels before and after open field or object recognition testing.Overview of c-Fos results for all brain areas investigated. Click here for file
