# Supplementary material for: Differential effects of diazepam and MPEP on habituation and neuro-behavioural processes in inbred mice
Source: Behav Brain Funct. 2012 Jun 11;8:30. doi: 10.1186/1744-9081-8-30 (PMC3464737; doi:10.1186/1744-9081-8-30)
Supplement: Additional file 1 — Schematic representations of the open field and the object recognition test.Corrected P value thresholds of significance for the open field and object recognition test.Overview of all behavioural results in the open field after MPEP or diazepam treatment.Overview of CORT levels before and after open field or object recognition testing.Overview of c-Fos results for all brain areas investigated. [file 1744-9081-8-30-S1.pdf]

# [Additional material]

### Additional figures

a)

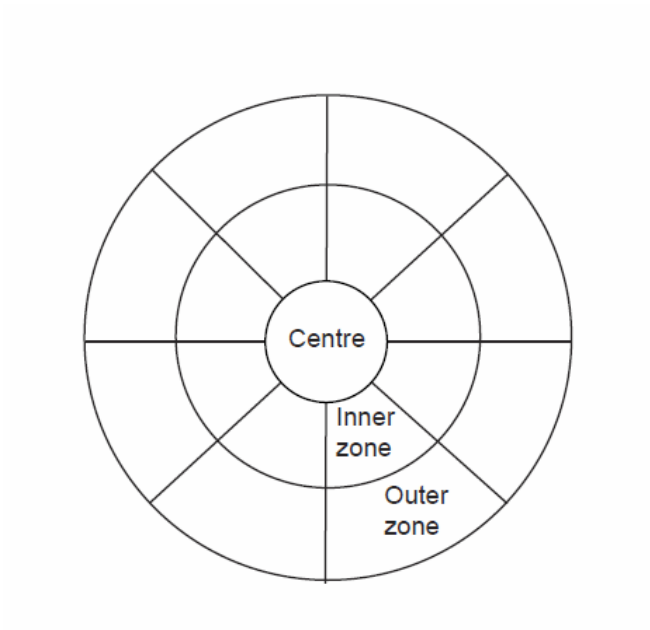


b)

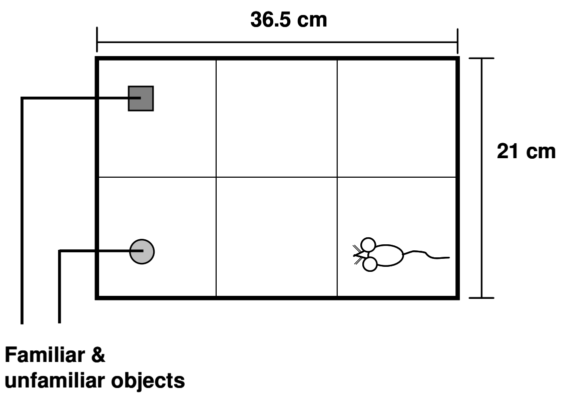


**Figure A1**: **Schematic drawing of the OF (a) and the ORT (b)**. a) Centre, inner and outer zone were divided by lines on the floor area. Extra lines were added to measure locomotor activity. b) After 24h familiarisation with one of the two objects, the familiar and an unfamiliar object were placed opposite the starting position.

**Table A1 Overview of the (corrected) thresholds of significance used in the statistical analyses in experiment 1, Open Field.**

| | Behavioural category/physiology/ c-Fos | Behavioural/physiological parameter/brain area | ANOVA * | *Post hoc* comparison # | Data can be found in |
|---|---|---|---|---|---|
| Behaviour | Avoidance behaviour | Latency until the first center entry | 0.0170 (q = 3) | 0.0043 (q = 12) | Table A3/A4 |
| | | Total time spent in center | | 0.0019 (q = 27) | |
| | | Total number of center entries | | 0.0019 (q = 27) | |
| | Risk assessment | Total number of stretched attends | 0.0253 (q = 2) | 0.0028 (q = 18) | |
| | | Latency until first stretched attend | | 0.0064 (q = 8) | |
| | Locomotor activity | Total number of line crossings | 0.0127 (q = 4) | 0.0014 (q = 36) | |
| | | Latency until first line crossing | | 0.0032 (q =16) | |
| | | Total time spent immobile | | 0.0014 (q = 36) | |
| | | Latency until the first immobility event | | 0.0032 (q =16) | |
| | Exploratory activity | Total number of rearings | 0.0253 (q = 2) | 0.0028 (q = 18) | |
| | | Latency until first rearing | | 0.0064 (q = 8) | |
| | Arousal | Total time spent grooming | 0.0170 (q = 3) | 0.0019 (q = 27) | |
| | | Latency until the first grooming event | | 0.0043 (q = 12) | |
| | | Total number of fecal boli | | 0.0043 (q = 12) | |
| Blood plasma | Stress response | Corticosterone | 0.0253 (q = 2) | 0.0052 (q = 10) | Table A5 |
| Brain | c-Fos | Medial prefrontal cortex | 0.05 (q = 1) | 0.0073 (q = 7) | Table A6 |
| | | Lateral septum | 0.0170 (q = 3) | 0.0024 (q = 21) | |
| | | Bed nucleus of the stria terminalis | 0.0170 (q = 3) | 0.0024 (q = 21) | |
| | | Hippocampus | 0.05 (q = 1) | 0.0073 (q = 7) | |
| | | Hypothalamus | 0.0253 (q = 2) | 0.0037 (q = 14) | |
| | | Amygdala | 0.0253 (q = 2) | 0.0037 (q = 14) | |
| | | Periaqueductal gray | 0.0127 (q = 4) | 0.0018 (q = 28) | |

* Calculating thresholds for the ANOVA: *i)* Behavioural parameters: $\alpha = 1 - [1 - 0.05]^{1/q}$; q = number of parameters per behavioural category. *ii*) Physiological parameters, α = 0.0253 (data from vehicle-treated animals are used twice). *iii)* Brain parameters: $\alpha = 1 - [1 - 0.05]^{1/q}$; q = number of subdivisions per brain area. The q values are shown in parentheses.
# Calculating thresholds for *post hoc* comparisons: *i)* Behavioural parameters: $\alpha = 1 - [1 - 0.05]^{1/q}$; q = number of parameters per behavioural category multiplied by the number of times a group is used for a meaningful comparison. *ii*) Physiological parameters, $\alpha = 1 - [1 - 0.05]^{1/q}$; q = number of meaningful comparisons between groups. *iii)* Brain parameters: $\alpha = 1 - [1 - 0.05]^{1/q}$; q = number of subdivisions per brain area multiplied by the times a group is used for meaningful comparisons.

**Table A2 Overview of the (corrected) thresholds of significance used in the statistical analyses in experiment 2, Object recognition test.**

| | Behavioural category/physiology | Behavioural/physiological parameter | ANOVA * | Post hoc comparison # | Data can be found in |
|---|---|---|---|---|---|
| Behaviour | Object memory | Discrimination index | 0.0102 (q = 5) | 0.0015 (q = 35) | Table A7 |
| | | Latency until first exploration novel object | | | |
| | | Latency until first exploration familiar object | | | |
| | | Total time spent exploring novel object | | | |
| | | Total time spent exploring familiar object | | | |
| | Risk assessment | Total number of stretched attends | 0.05 (q = 1) | 0.0073 (q = 7) | |
| | Locomotion | Total number of line crossings | 0.0170 (q = 3) | 0.0024 (q = 21) | |
| | | Total time spent immobile | | | |
| | | Latency until the first immobility event | | | |
| | General exploration | Total number of rearings | 0.05 (q = 1) | 0.0073 (q = 7) | |
| | Arousal/de-arousal | Total time spent grooming | | | |
| | | Latency until the first grooming event | 0.0253 (q = 2) | 0.0037 (q = 14) | |
| Blood plasma | Stress response | Corticosterone | 0.05 (q = 1) | 0.0052 (q = 10) | Table A8 |

* Calculating thresholds for the ANOVA: *i)* Behavioural parameters: $\alpha = 1 - [1 - 0.05]^{1/q}$; q = number of parameters per behavioural category. *ii*) Physiological parameters, $\alpha = 0.05$.

# Calculating thresholds for *post hoc* comparisons: *i)* Behavioural parameters: $\alpha = 1 - [1 - 0.05]^{1/q}$; q = number of parameters per behavioural category multiplied by the number of times a group is used for a meaningful comparison. *ii*) Physiological parameters, $\alpha = 1 - [1 - 0.05]^{1/q}$; q = number of meaningful comparisons between groups.

**Table A3 Overview of behavioural parameters measured in the OF after vehicle, 1 mg/kg, 3 mg/kg or 5 mg/kg diazepam**. The 30 min OF trial was divided in 6 time intervals of 5 min each. Data are shown per time interval, only latency and defecation data are shown once per OF trial.

| Behavioural parameter | Time interval ‡ | ANOVA effects @ | vehicle | | 1 mg/kg diazepam | | 3 mg/kg diazepam | | 5 mg/kg diazepam | |
|---|---|---|---|---|---|---|---|---|---|---|
| | | | BALB/c | 129P3 | BALB/c | 129P3 | BALB/c | 129P3 | BALB/c | 129P3 |
| Latency centre | 1-6 | ns | 684.9 ± 221.2 | 487.1 ± 228.5 | 311.5 ± 215.0 | 89.6 ± 21.7 | 395.9 ± 130.8 | 598.4 ± 265.3 | 274.0 ± 119.0 | 248.1 ± 107.8 |
| Centre duration | 1 | T | 0.8 ± 0.7 | 2.0 ± 0.9 | 1.8 ± 0.7 | 1.8 ± 0.6 | 0.9 ± 0.6 | 0.5 ± 0.3 | 0.5 ± 0.2 | 0.5 ± 0.2 |
| | 2 | | 1.2 ± 0.5 | 1.2 ± 2.4 | 1.4 ± 0.5 | 1.7 ± 0.4 | 0.7 ± 0.3 | 3.1 ± 2.7 | 0.7 ± 0.3 | 7.9 ± 6.3 |
| | 3 | | 2.0 ± 0.7 | 1.6 ± 0.7 | 2.3 ± 0.7 | 1.8 ± 0.6 | 1.3 ± 0.3 | 2.0 ± 1.8 | 1.1 ± 0.5 | 1.0 ± 0.6 |
| | 4 | | 3.0 ± 0.8 | 2.1 ± 0.9 | 1.9 ± 0.9 | 2.5 ± 1.1 | 2.0 ± 0.6 | 0.4 ± 0.2 | 1.7 ± 0.7 | 0.4 ± 0.2 |
| | 5 | | 2.6 ± 0.8 | 2.8 ± 1.1 | 3.3 ± 1.4 | 2.6 ± 0.6 | 2.1 ± 0.6 | 0.3 ± 0.1 | 1.9 ± 0.6 | 1.7 ± 0.8 |
| | 6 | | 2.3 ± 0.6 | 3.1 ± 1.1 | 1.6 ± 0.6 | 1.9 ± 0.8 | 2.0 ± 0.5 | 0.2 ± 0.1 | 1.3 ± 0.4 | 0.8 ± 0.4 |
| Centre entries | 1 | T, T*S | 1 (2) | 3 (6) | 5 (6) | 3 (2) | 1 (3) | 1 (3) | 2 (3) | 2 (2) |
| | 2 | | 2 (7) | 3 (5) | 5 (6) | 5 (4) | 2 (4) | 2 (1) | 3 (3) | 2 (2) |
| | 3 | | 8 (8) | 4 (5) | 8 (9) | 2 (1) | 3 (3) | 0 (1) | 3 (4) | 0 (2) |
| | 4 | | 8 (9) | 4 (6) | 5 (4) | 3 (3) | 6 (8) | 1 (1) | 4 (3) | 1 (1) |
| | 5 | | 6 (5) | 5 (5) | 9 (5) | 4 (3) | 9 (7) | 0 (1) | 4 (2) | 1 (1) |
| | 6 | | 7 (6) † | 4 (4) | 5 (6) | 5 (1) | 7 (7) | 1 (1) | 3 (3) | 1 (2) |
| Stretched attends | 1 | T, D, T*D | 18 (22 ) | 14 (35) | 5 (6) | 7 (6) | 5 (7) | 6 (7) | 4 (2) | 1 (3) |
| | 2 | | 4 (12) | 2 (6) | 0 (2) | 0 (1) | 0 (1) | 0 (1) | 0 (0) † | 0 (1) |
| | 3 | | 2 (5) | 0 (2) | 0 (0) | 0 (0) | 0 (0) † | 0 (3) | 0 (0) † | 0 (2) |
| | 4 | | 1 (4) | 0 (2) | 0 (0) † | 0 (0) | 0 (0) † | 0 (3) | 0 (0) † | 0 (0) |
| | 5 | | 0 (3) | 0 (1) | 0 (0) † | 0 (0) | 0 (0) † | 0 (0) | 0 (0) † | 0 (0) |
| | 6 | | 0 (0) † | 0 (0) † | 0 (1) † | 0 (0) | 0 (0) † | 0 (1) | 0 (0) † | 0 (0) |
| Latency stretched attend | 1-6 | ns | 16.5 ± 3.9 | 231.2 ± 224.1 | 8.8 ± 1.9 | 231.0 ± 224.1 | 291.0 ± 222.2 | 263.2 ± 165.3 | 19.1 ± 8.9 | 244.5 ± 222.3 |
| Line crossings | 1 | S, T*S | 146 (153) | 105 (153) | 286 (104) | 181 (44) | 134 (89) | 53 (153) | 217 (82) | 41 (104) |
| | 2 | | 171 (107) | 120 (78) | 298 (90) | 133 (64) | 128 (139) | 33 (57) | 187 (82) | 13 (77) ^ |
| | 3 | | 213 (105) | 123 (99) | 268 (76) | 114 (60) | 200 (131) | 23 (51) | 176 (44) | 30 (51) |
| | 4 | | 166 (133) | 100 (76) | 287 (71) | 137 (72) | 251 (173) | 50 (73) | 192 (54) | 60 (47) |
| | 5 | | 221 (103) | 122 (77) | 256 (94) | 118 (95) | 333 (106) | 39 (55) ^ | 213 (49) | 59 (45) ^ |
| | 6 | | 230 (75) † | 116 (86) ^ | 287 (140) | 117 (73) | 363 (163) † | 14 (52) ^ | 240 (87) | 51 (48) ^ |
| Latency line cross | 1-6 | ns | 45.8 ± 11.0 | 11.8 ± 3.0 | 13.0 ± 3.1 | 8.1 ± 1.1 | 18.0 ± 6.0 | 35.1 ± 6.8 | 19.0 ± 3.6 | 33.2 ± 17.0 |
| Immobility duration | 1 | S, D, S*D | 5.0 ± 4.3 | 0.3 ± 0.3 | 0.0 ± 0.0 | 0.6 ± 0.3 | 5.7 ± 2.9 | 30.1 ± 10.8 ^* | 7.7 ± 5.0 | 32.6 ± 9.2 ^* |
| | 2 | | 0.3 ± 0.2 | 2.1 ± 1.8 | 0.6 ± 0.6 | 2.2 ± 1.1 | 6.2 ± 5.2 | 32.5 ± 11.1 ^* | 6.4 ± 3.8 | 45.2 ± 13.8 ^* |
| | 3 | | 0.0 ± 0.0 | 1.3 ± 0.7 | 0.4 ± 0.3 | 1.5 ± 1.0 | 10.2 ± 9.2 | 44.3 ± 11.6 | 5.3 ± 3.6 | 40.7 ± 13.2 ^* |
| | 4 | | 5.6 ± 5.6 | 3.2 ± 2.5 | 0.4 ± 0.3 | 1.6 ± 1.0 | 2.7 ± 1.9 | 32.2 ± 12.1 ^* | 1.3 ± 1.2 | 33.8 ± 13.2 ^* |
| | 5 | | 1.2 ± 0.8 | 0.6 ± 0.4 | 0.4 ± 0.4 | 2.5 ± 1.3 | 0.8 ± 0.6 | 27.7 ± 9.9 ^* | 2.4 ± 2.4 | 24.5 ± 10.4 ^* |
| | 6 | | 0.5 ± 0.3 | 5.3 ± 4.9 | 0.4 ± 0.4 | 0.6 ± 0.4 | 4.1 ± 4.0 | 38.5 ± 12.1 | 4.2 ± 2.7 | 33.5 ± 11.5 |
| Latency immobility | 1-6 | D | 778.2 ± 240.9 | 830.7 ± 212.6 | 1286.3 ± 34.7 | 600.7 ± 217.4 | 300.9 ± 218.1 | 343.6 ± 22.9* | 596.7 ± 231.2 | 141.5 ± 60.9 * |
| Rearings | 1 | T, D. T*D | 2 (3) | 2 (9) | 6 (17) | 5 (6) | 1 (3) | 0 (0) | 1 (2) | 1 (1) |
| | 2 | | 3 (21) | 9 (17) | 27 (46) | 10 (12) | 2 (11) | 1 (2) | 3 (9) | 0 (2) |
| | 3 | | 19 (34) | 9 (10) | 43( 40) † | 21 (17) | 4 (22) | 1 (3) | 5 (9) | 1 (4) |
| | 4 | | 26 (30) | 16 (16) | 33 (45) † | 25 (25) | 6 (14) | 2 (4) | 7 (5) | 2 (4) |
| | 5 | | 32 (30) † | 15 (17) | 49 (28) † | 27 (33) | 6 (23) | 2 (4) | 7 (6) | 2 (12) |
| | 6 | | 37 (36) † | 16 (23) | 45 (43) † | 31 (9) | 9 (21) | 2 (10) * | 13 (15) | 2 (11) * |
| Latency rearing | 1-6 | ns | 349.9 ± 102.1 | 263.9 ± 108.3 | 341.6 ± 140.1 | 127.4 ± 22.6 | 300.5 ± 55.9 | 795.2 ± 242.7 | 337.9 ± 72.6 | 678.5 ± 210.7 |

**[ Table A3 continues on next page ]**

**[ Table A3 continued ]**

| | | | | | | | | | | |
|---|---|---|---|---|---|---|---|---|---|---|
| Grooming duration | 1 | ns | 1.5 ± 0.5 | 1.6 ± 0.6 | 1.6 ± 1.3 | 0.0 ± 0.0 | 0.4 ± 0.2 | 0.3 ± 0.3 | 2.8 ± 1.3 | 2.9 ± 0.9 |
| | 2 | | 2.8 ± 1.3 | 3.9 ± 1.2 | 0.3 ± 0.3 | 3.5 ± 2.9 | 5.0 ± 2.4 | 6.0 ± 1.5 | 6.8 ± 3.8 | 6.1 ± 2.4 |
| | 3 | | 4.2 ± 1.4 | 3.1 ± 1.7 | 2.4 ± 1.7 | 4.9 ± 3.9 | 1.8 ± 0.7 | 11.3 ± 6.3 | 7.2 ± 4.1 | 11.2 ± 3.5 |
| | 4 | | 3.7 ± 1.3 | 5.7 ± 2.3 | 0.3 ± 0.2 | 3.1 ± 2.0 | 2.0 ± 0.4 | 9.4 ± 2.9 | 6.0 ± 2.6 | 9.9 ± 3.5 |
| | 5 | | 2.4 ± 0.9 | 3.7 ± 2.5 | 0.5 ± 0.3 | 3.4 ± 2.4 | 6.4 ± 1.2 † | 4.6 ± 1.3 | 4.9 ± 1.7 | 6.3 ± 2.3 |
| | 6 | | 2.4 ± 1.0 | 5.3 ± 2.2 | 0.1 ± 0.1 | 3.8 ± 3.2 | 6.7 ± 2.4 | 10.4 ± 3.3 | 8.2 ± 1.9 | 7.7 ± 2.5 |
| Latency self-groom | 1-6 | ns | 372.9 ± 56.2 | 443.5 ± 73.0 | 652.1 ± 135.7 | 439.6 ± 41.0 | 521.6 ± 172.2 | 754.7 ± 213.2 | 393.4 ± 83.6 | 757.1 ± 181.6 |
| Defecation | 1-6 | D | 13 (3) | 8 (2) | 5 (7) | 9 (4) | 4 (2) * | 5 (4) | 9 (5) | 4 (5) |

Data are represented as mean ± SEM for continuous numerical data, for discrete numerical data on the ordinal scale the results are represented as median with in parentheses IQR. Multivariate repeated measures ANOVA was performed with time interval as within factor and strain and dose as between factor. Latencies and defecation parameter were analyzed with a two-way ANOVA with strain and dose as main factors.

‡ The open field test of 30 minutes was divided in 6 bins of 5 minutes each (time interval). Number and duration of a specific behavioural parameter were calculated per time interval except for the latency and defecation, which is given only once per whole trial.

@ Significant ANOVA effects: S = strain effect; T = time interval effect; D = dose effect; T*S interaction; T*D interaction; S*D interaction; ns = non-significant. Thresholds of significance used for the ANOVA analyses, according to the Dunn- Šidák correction for multiple comparisons (see Experimental procedures 2.8 and Table A1 for the corrected P values).

Significant *post hoc* differences are shown as: * = vs. vehicle, ^ = vs. BALB/c, † = vs. time interval 1. Thresholds of significance used for *post hoc* comparisons can also be found in Table A1

**Table A4 Overview of behavioural parameters measured in the OF after vehicle, 3 mg/kg, 10 mg/kg or 30 mg/kg MPEP**. For behavioural analyses, the 30 min OF trial was divided in 6 time intervals of 5 min each. Data are shown per time interval, only latency and defecation data are shown once per OF trial.

| Behavioural parameter | Time interval ‡ | ANOVA effects @ | vehicle | | 3 mg/kg MPEP | | 10 mg/kg MPEP | | 30 mg/kg MPEP | |
|---|---|---|---|---|---|---|---|---|---|---|
| | | | BALB/c | 129P3 | BALB/c | 129P3 | BALB/c | 129P3 | BALB/c | 129P3 |
| Latency centre | 1-6 | S | 684.9 ± 221.2 | 487.1 ± 228.5 | 453.6 ± 154.9 | 565.5 ± 272.0 | 1250.5 ± 106 | 184.5 ±120.3^ | 525.5 ±279.0 | 125.1 ± 39.4^ |
| Centre duration | 1 | T | 0.8 ± 0.7 | 2.0 ± 0.9 | 0.7 ± 0.4 | 1.4 ± 0.6 | 0.6 ± 0.4 | 2.1 ± 0.6 | 1.9 ± 0.9 | 1.1 ± 0.2 |
| | 2 | | 1.2 ± 0.5 | 1.2 ± 2.4 | 0.6 ± 0.3 | 1.6 ± 0.6 | 1.0 ± 0.7 | 2.9 ± 0.6 | 2.5 ± 1.6 | 3.2 ± 0.5 |
| | 3 | | 2.0 ± 0.7 | 1.6 ± 0.7 | 0.8 ± 0.4 | 1.9 ± 1.1 | 0.6 ± 0.4 | 2.6 ± 0.8 | 1.9 ± 0.8 | 3.2 ± 0.5 |
| | 4 | | 3.0 ± 0.8 | 2.1 ± 0.9 | 3.5 ± 1.0 | 2.0 ± 0.6 | 1.2 ± 0.9 | 3.0 ± 0.6 | 3.5 ± 1.0 | 4.2 ± 1.1 |
| | 5 | | 2.6 ± 0.8 | 2.8 ± 1.1 | 3.8 ± 1.0 | 2.0 ± 0.7 | 1.4 ± 0.8 | 3.6 ± 1.3 | 2.4 ± 0.7 | 2.8 ± 0.5 |
| | 6 | | 2.3 ± 0.6 | 3.1 ± 1.1 | 3.0 ± 0.7 | 2.6 ± 1.6 | 1.4 ± 0.8 | 2.1 ± 0.5 | 3.2 ± 1.2 | 2.6 ± 0.5 |
| Centre entries | 1 | T | 1 (2) | 3 (6) | 0 (4) | 2 (3) | 0 (1) | 6 (4) | 3 (5) | 5 (4) |
| | 2 | | 2 (7) | 3 (5) | 0 (2) | 3 (4) | 0( 3) | 7 (2) | 3 (7) | 9 (4) |
| | 3 | | 8 (8) | 4 (5) | 2 (3) | 2 (6) | 0 (3) | 7 (4) | 5 (7) | 12 (6) |
| | 4 | | 8 (9) | 4 (6) | 4 (3) | 5 (8) | 0 (4) | 8 (3) | 7 (11) | 10 (4) |
| | 5 | | 6 (5) | 5 (5) | 6 (4) | 5 (7) | 1 (5) | 6 (2) | 5 (8) | 8 (4) |
| | 6 | | 7 (6) | 4 (4) | 6 (2) | 4 (9) | 2 (6) | 5 (3) | 8 (4) | 9 (2) |
| Stretched attends | 1 | T, T*S | 18 (22 ) | 14 (35) | 26 (21) | 11 (20) | 23 (14) | 5 (10) | 16 (31) | 4 (3) ^ |
| | 2 | | 4 (12) | 2 (6) | 5 (8) | 2 (16) | 9 (13) | 0 (0) | 3 (15) | 0 (0) |
| | 3 | | 2 (5) | 0 (2) | 0 (2) | 1 (9) | 10 (15) | 0 (0) ^ | 1 (5) | 0 (0) |
| | 4 | | 1 (4) | 0 (2) | 0 (2) | 0 (3) | 10 (14) | 0 (0) ^ | 0 (5) | 0 (0) |
| | 5 | | 0 (3) † | 0 (1) | 0 (0) | 0 (10) | 5 (11) | 0 (0) | 1 (4) | 0 (0) |
| | 6 | | 0 (0) † | 0 (0) † | 0 (0) | 0 (8) | 0 (7) | 0 (0) | 0 (5) | 0 (0) |
| Latency stretched attend | 1-6 | ns | 16.5 ± 3.9 | 231.2 ± 224.1 | 10.2 ± 2.7 | 230.6 ± 224.2 | 5.8 ± 1.0 | 231.8 ± 224.0 | 232.0 ±224.0 | 231.6 ± 224.1 |
| Line crossings | 1 | T*S | 146 (153) | 105 (153) | 105 (68) | 140 (210) | 89 (115) | 199 (46) | 189 (127) | 204 (42) |
| | 2 | | 171 (107) | 120 (78) | 104 (53) | 135 (141) | 93 (86) | 159 (36) | 169 (99) | 183 (64) |
| | 3 | | 213 (105) | 123 (99) | 129 (56) | 137 (68) | 114 (58) | 144 (63) | 174 (90) | 152 (37) |
| | 4 | | 166 (133) | 100 (76) | 177 (50) | 145 (82) | 118 (104) | 127 (58) | 205 (121) | 156 (50) |
| | 5 | | 221 (103) | 122 (77) | 166 (64) | 137 (70) | 131 (128) | 130 (59) | 149 96) | 159 (24) |
| | 6 | | 230 (75) † | 116 (86) | 192 (35) | 133 (96) | 174 (129) | 131 (63) | 221 (130) | 138 (52) |
| Latency line cross | 1-6 | S | 45.8 ± 11.0 | 11.8 ± 3.0 | 16.5 ± 3.2 | 21.0 ± 8.3 | 29.3 ± 9.9 | 5.4 ± 1.3 | 12.4 ± 2.3 | 7.3 ± 1.1 |
| Immobility duration | 1 | ns | 5.0 ± 4.3 | 0.3 ± 0.3 | 1.8 ± 1.8 | 5.6 ± 2.7 | 0.0 ± 0.0 | 0.0 ± 0.0 | 0.2 ± 0.2 | 0.4 ± 0.4 |
| | 2 | | 0.3 ± 0.2 | 2.1 ± 1.8 | 0.3 ± 0.3 | 4.0 ± 1.9 | 0.2 ± 0.2 | 0.1 ± 0.1 | 0.0 ± 0. | 0.5 ± 0.5 |
| | 3 | | 0.0 ± 0.0 | 1.3 ± 0.7 | 2.0 ± 1.3 | 5.4 ± 3.4 | 0.4 ± 0.4 | 0.0 ± 0.0 | 0.1 ± 0.3 | 0.2 ± 0.1 |
| | 4 | | 5.6 ± 5.6 | 3.2 ± 2.5 | 2.2 ± 2.2 | 5.9 ± 4.2 | 0.4 ± 0.4 | 0.1 ± 0.1 | 0.6 ± 0.4 | 0.2 ± 0.1 |
| | 5 | | 1.2 ± 0.8 | 0.6 ± 0.4 | 0.4 ± 0.4 | 1.8 ± 0.8 | 1.1 ± 0.8 | 0.3 ± 0.3 | 0.2 ± 0.2 | 0.1 ± 0.1 |
| | 6 | | 0.5 ± 0.3 | 5.3 ± 4.9 | 0.3 ± 0.3 | 3.0 ± 1.9 | 1.5 ± 1.1 | 0.3 ± 0.3 | 0.4 ± 0.2 | 0.0 ± 0.0 |
| Latency immobility | 1-6 | D | 778.2 ± 240.9 | 830.7 ± 212.6 | 1087.3 ± 51.2 | 526.0 ± 206.0 | 1559.2 ±64.8* | 1531.7±175.7* | 832.9 ± 12.6 | 1070.6 ± 248.7 |
| Rearings | 1 | T | 2 (3) | 2 (9) | 1 (2) | 5 (6) | 2 (4) | 4 (4) | 2 (14) | 6 (9) |
| | 2 | | 3 (21) | 9 (17) | 6 (8) | 12 (11) | 1 (6) | 10 (11) | 10 (17) | 7 (8) |
| | 3 | | 19 (34) | 9 (10) | 12 (14) | 16 (15) | 4 (13) | 17 (9) | 11 (18) | 14 (15) |
| | 4 | | 26 (30) | 16 (16) | 18 (14) | 15 (17) | 7 (16) | 13 (9) | 28 (27) | 18 (22) |
| | 5 | | 32 (30) † | 15 (17) | 32 (14) | 25 (31) | 9 (20) | 28 (3) † | 22 (22) | 18 (21) |
| | 6 | | 37 (36) † | 16 (23) | 52 (16) † | 20 (43) | 12 (27) | 27 (22) † | 30 (17) | 17 (16) † |
| Latency rearing | 1-6 | S | 349.9 ± 102.1 | 263.9 ± 108.3 | 269.0 ± 72.9 | 233.5 ± 110.9 | 635.9 ± 262.4 | 234.2 ± 102.4 | 151.2 ± 51.4 | 200.5 ± 107.8 |

**[ Table A4 continues on next page ]**

**[ Table A4 continued ]**

| | | | | | | | | | | |
|---|---|---|---|---|---|---|---|---|---|---|
| Grooming duration | 1 | S, D | 1.5 ± 0.5 | 1.6 ± 0.6 | 2.8 ± 1.5 | 9.5 ± 3.3 | 9.7 ± 5.7 | 1.9 ± 0.6 | 1.0 ± 0.4 | 4.0 ± 1.2 |
| | 2 | | 2.8 ± 1.3 | 3.9 ± 1.2 | 1.6 ± 0.6 | 5.3 ± 1.7 | 5.4 ± 1.6 | 10.6 ± 4.2 | 7.6 ± 2.2 | 3.1 ± 1.4 |
| | 3 | | 4.2 ± 1.4 | 3.1 ± 1.7 | 6.0 ± 2.8 | 7.4 ± 2.3 | 0.7 ± 0.4 | 8.9 ± 3.6 | 5.9 ± 1.0 | 10.5 ± 3.3 |
| | 4 | | 3.7 ± 1.3 | 5.7 ± 2.3 | 1.3 ± 0.6 | 10.0 ± 4.4 | 12.1 ± 4.9 | 8.9 ± 2.1 | 5.5 ± 2.0 | 8.6 ± 3.2 |
| | 5 | | 2.4 ± 0.9 | 3.7 ± 2.5 | 3.7 ± 1.3 | 9.7 ± 4.9 | 4.0 ± 1.9 | 6.1 ± 3.4 | 11.0 ± 4.4 | 12.4 ± 3.5 |
| | 6 | | 2.4 ± 1.0 | 5.3 ± 2.2 | 2.8 ± 1.0 | 4.3 ± 1.9 | 5.9 ± 2.5 | 5.5 ± 1.6 | 3.6 ± 1.3 | 10.2 ± 3.8 |
| Latency self-groom | 1-6 | ns | 372.9 ± 56.2 | 443.5 ± 73.0 | 479.5 ± 119.0 | 539.5 ± 44.9 | 415.0 ± 47.0 | 448.0 ± 62.3 | 414.7 ± 33.3 | 226.1 ± 38.4 |
| Defecation | 1-6 | S | 13 (3) | 8 (2) | 7 (4) | 8 (7) | 10 (4) | 8 (2) | 12 (9) | 6 (3) ^ |

Data are represented as mean ± SEM for continuous numerical data, for discrete numerical data on the ordinal scale the results are represented as median with in parentheses IQR. Multivariate repeated measures ANOVA was performed with time interval as within factor and strain and dose as between factor. Latencies and defecation parameter were analyzed with a two-way ANOVA with strain and dose as main factors.

‡ The open field test of 30 minutes was divided in 6 bins of 5 minutes each (time interval). Number and duration of a specific behavioural parameter were calculated per time interval except for the latency and defecation, which is given only once per whole trial.

@ Significant ANOVA effects: S = strain effect; T = time interval effect; D = dose effect; T*S interaction; T*D interaction; S*D interaction; ns = non-significant. Thresholds of significance used for the ANOVA analyses, according to the Dunn- Šidák correction for multiple comparisons (see Experimental procedures 2.8 and Table A1 for the corrected P values).

Significant *post hoc* differences are shown as: * = vs. vehicle, ^ = vs. BALB/c, † = vs. time interval 1. Thresholds of significance used for *post hoc* comparisons can also be found in Table A1

**Table A5: Overview of CORT levels before (basal) and after behavioural testing (non-basal) in the OF after diazepam or MPEP treatment.**

| | | ANOVA effects @ | BALB/c basal | BALB/c non-basal | 129P3 basal | 129P3 non-basal |
|---|---|---|---|---|---|---|
| diazepam | vehicle | B, S, D, S*D | 191.8 ± 27.4 | 391.5 ± 35.4 * | 265.3 ± 45.0 | 445.9 ± 29.5 * |
| | 1 mg/kg diazepam | | 245.8 ± 57.5 | 372.3 ± 34.8 | 221.4 ± 67.9 | 253.3 ± 54.4 |
| | 3 mg/kg diazepam | | 282.6 ± 53.6 | 263.8 ± 32.4 | 163.7 ± 7.8a | 551.1 ± 128.2 * |
| | 5 mg/kg diazepam | | 183.6 ± 30.4 | 211.3 ± 34.6b | 232.3 ± 36.9a | 589.5 ± 100.7 # * |
| MPEP | vehicle | B, S | 191.8 ± 27.4 | 391.5 ± 35.4 * | 265.3 ± 45.0 | 445.9 ± 29.5 * |
| | 3 mg/kg MPEP | | 159.6 ± 22.3 | 398.0 ± 44.6 * | 293.0 ± 53.8 | 446.8 ± 85.0 |
| | 10 mg/kg MPEP | | 179.4 ± 26.7 | 353.0 ± 37.8 * | 293.7 ± 75.5 | 375.4 ± 82.5 |
| | 30 mg/kg MPEP | | 131.8 ± 10.6 | 425.3 ± 81.6 * | 194.3 ± 30.3 | 460.3 ± 110.7 |

Data are presented as mean nmol/l ± SEM. Multivariate repeated measures ANOVA was performed with basal/non-basal as within factor and strain and dose as between factor.

@ Significant ANOVA effects: B = basal/non-basal effect; S = strain effect; D = dose effect; S*D interaction. Threshold of significance used for the ANOVA analyses, according to the Dunn- Šidák correction for multiple comparisons (see Experimental procedures 2.8 and Table A1 for the corrected P values)

Significant *post hoc* effects are shown as: * = vs. vehicle, # = vs. BALB/c. Thresholds of significance used for *post hoc* comparisons can also be found in Table A1.

**Table A6: Overview of c-Fos positive cells after behavioural testing in the OF after treatment with vehicle, 1 mg/kg diazepam or 10 mg/kg MPEP.**

| Brain area | subdivision | ANOVA effects @ | BALB/c vehicle | BALB/c 1 mg/kg diazepam | BALB/c 10 mg/kg MPEP | 129P3 vehicle | 129P3 1 mg/kg diazepam | 129P3 10 mg/kg MPEP |
|---|---|---|---|---|---|---|---|---|
| Medial prefrontal cortex | PrL | S, Tr, S*Tr | 157.8 ± 18.5 | 97.9 ± 6.8 * | 93.6 ± 17.6 * | 65.4 ± 14.9 # | 47.4 ± 11.2 # | 105.7 ± 11.3 * |
| Lateral septum | LSD | S, Tr | 47.6 ± 10.8 | 18.8 ± 10.8 | 21.9 ± 9.9 | 11.8 ± 7.4 # | 4.0 ± 3.0 | 6.3 ± 2.3 |
| | LSI | ns | 35.4 ± 9.7 | 22.0 ± 5.4 | 20.4 ± 3.8 | 19.1 ± 5.7 | 18.4 ± 4.9 | 16.9 ± 4.5 |
| | LSV | Tr | 64.9 ± 20.2 | 26.6 ± 4.5 | 22.9 ± 7.6 * | 48.4 ± 14.8 | 36.9 ± 15.3 | 17.7 ± 9.5 * |
| Bed nucleus stria terminalis | BSTMA | ns | 62.3 ± 7.6 | 37.9 ± 12.2 | 67.5 ± 11.0 | 40.6 ± 7.7 | 48.8 ± 5.6 | 82.9 ± 14.3 |
| | BSTLP | S*Tr | 62.6 ± 11.1 | 38.5 ± 6.2 | 51.0 ± 8.0 | 29.0 ± 5.0 | 58.4 ± 10.5 | 71.6 ± 11.1 * |
| | BSTMV | ns | 23.2 ± 2.9 | 27.6 ± 6.2 | 22.9 ± 5.2 | 19.8 ± 5.4 | 30.6 ± 6.6 | 58.0 ± 18.0 |
| Hippocampus | DG | S | 72.3 ± 12.2 | 73.2 ± 10.9 | 73.8 ± 10.2 | 25.0 ± 6.0 # | 25.5 ± 4.3 # | 48.2 ± 14.7 |
| Hypothalamus | PVN | S, Tr , S*Tr | 101.3 ± 20.6 | 44.8 ± 8.5 * | 75.9 ± 11.4 | 120.5 ± 20.7 | 58.0 ± 16.0 | 20.6 ± 5.8 # * |
| | DMH | S, Tr | 138.8 ± 33.4 | 114.9 ± 19.4 | 53.9 ± 17.4 * | 89.9 ± 8.0 | 61.5 ± 8.2 * | 40.0 ± 5.8 * |
| Amygdala | BLA | S*Tr | 51.9 ± 9.1 | 39.2 ± 5.9 * | 41.1 ± 5.2 | 42.9 ± 4.9 | 18.1 ± 2.6 * | 31.3 ± 3.2 * |
| | cAmy | ns | 35.5 ± 8.6 | 34.2 ± 5.8 | 28.8 ± 8.0 | 40.7 ± 5.6 | 18.7 ± 3.4 | 21.9 ± 4.7 |
| Periaqueductal gray | dlPAG | ns | 119.9 ± 13.9 | 77.6 ± 8.9 | 56.0 ± 14.1 | 63.4 ± 10.3 | 63.9 ± 7.9 | 69.9 ± 6.7 |
| | dmPAG | ns | 2.2 ± 26.3 | 57.7 ± 22.1 | 31.8 ± 12.7 | 35.8 ± 14.0 | 52.6 ± 18.9 | 43.5 ± 15.2 |
| | lPAG | ns | 108.9 ± 14.1 | 85.7 ± 12.3 | 66.1 ± 17.1 | 106.2 ± 19.9 | 105.5 ± 13.8 | 76.7 ± 4.5 |
| | vlPAG | Tr | 85.4 ± 13.7 | 60.2 ± 5.8 | 46.9 ± 7.0 * | 98.9 ± 21.9 | 70.6 ± 7.1 | 56.5 ± 4.4 * |

Abbreviations: PrL (prelimbic cortex), LSD (dorsal lateral septum), LSI (intermediary lateral septum), V (ventral lateral septum), BSTMA (bed nucleus of the stria terminalis, medial anterior part), BSTLP (bed nucleus of the stria terminalis, lateral posterior part), BSTMV (bed nucleus of the stria terminalis, medial ventral part), DG (dentate gyrus), PVN (paraventricular nucleus), DMH (dorsal medial hypothalamus), BLA (basolateral amygdala), cAmy (central nucleus of the amygdala), dlPAG (dorsolateral part of periaqueductal grey), dmPAG (dorsomedial part of the periaqueductal grey), lPAG (lateral part of the periaqueductal grey) and the vlPAG (ventrolateral part of the periaqueductal grey).

Results are represented as the mean number of c-Fos positive cells per $mm^2$ (± SEM). A two-way ANOVA was performed with strain and treatment as main factors.

@ Significant ANOVA effects: S = strain effect; Tr = treatment effect; S*Tr interaction effect; ns = non-significant. Thresholds of significance used for ANOVA analyses, according to the Dunn- Šidák correction for multiple comparisons (see Experimental procedures 2.8 and Table A1 for the corrected P values).

Significant *post hoc* differences are shown as: * = vs. vehicle # = vs. BALB/c. Thresholds of significance used for *post hoc* comparisons can also be found in Table A1.

**Table A7**: **Overview of behavioural parameters measured in the object recognition test.**

| | | BALB/c | | | 129P3 | | |
|---|---|---|---|---|---|---|---|
| | ANOVA effects @ | vehicle | diazepam | MPEP | vehicle | diazepam | MPEP |
| Discrimination Index (DI) | S | 0.28 ± 0.06 | 0.20 ± 0.08 | 0.31 ± 0.04 | -0.05 ± 0.15 # | 0.06 ± 0.05 | 0.16 ± 0.08 |
| Latency novel object [sec] | ns | 23.1 ± 4.4 | 21.2 ± 2.6 | 51.4 ± 8.1 | 18.0 ± 3.3 | 24.8 ± 5.2 | 28.5 ± 4.7 |
| Latency familiar object [sec] | ns | 23.5 ± 5.1 | 18.9 ± 2.4 | 43.7 ± 9.6 | 23.8 ± 5.4 | 31.4 ± 5.6 | 32.3 ± 6.8 |
| Time novel object [%] | ns | 7.1 ± 1.3 | 5.8 ± 1.2 | 5.5 ± 0.9 | 4.3 ± 0.9 | 5.9 ± 0.7 | 5.6 ± 0.6 |
| Time familiar object [%] | ns | 4.0 ± 0.7 | 4.1 ± 0.9 | 2.8 ± 0.4 | 4.1 ± 0.5 | 5.0 ± 0.4 | 4.1 ± 0.5 |
| Stretched attends [nr] | Tr | 5.5 (4.5) | 6.0 (6.5) | 17.5 (12.5) | 11.0 (11.0) | 12.0 (5.5) | 5.0 (6.5) |
| Line crossings [nr] | S | 159.5 (68.0) | 176.0 (35.5) | 149.5 (38.5) | 91.0 (94.0) # | 60.0 (67.5) # | 69.0 (56.0) # |
| Immobility duration [%] | S | 0.5 ± 0.3 | 0.3 ± 0.1 | 0.3 ± 0.2 | 11.8 ± 4.5 # | 23.4 ± 5.4 # | 11.7 ± 3.4 # |
| Latency immobility | S | 425.7 ± 75.5 | 405.3 ± 76.5 | 479.8 ± 74.5 | 169.7 ± 37.3 # | 140.3 ± 29.8 # | 190.7 ± 30.4 # |
| Rearings [nr] | S | 85.5 (30.5) | 126.0 (38.0) | 77.5 (74.0) | 30.0 (75.0) | 10.0 (27.5) | 19.0 (28.0) |
| Grooming [%] | ns | 2.2 ± 0.5 | 1.3 ± 0.4 | 1.6 ± 0.4 | 2.1 ± 0.4 | 1.1 ± 0.3 | 1.8 ± 0.3 |
| Latency groom [sec] | Tr | 189.5 ± 20.4 | 300.9 ± 37.5 * | 172.7 ± 11.2 | 223.7 ± 44.4 | 302.6 ± 41.4 * | 188.2 ± 27.7 * |

Data are presented as mean ± SEM for continuous data, for discrete data on the ordinal scale the results are presented as median with in parentheses IQR. Two-way ANOVA was performed using strain and treatment as main factors.

@ Significant ANOVA effects: S = strain effect; T = time interval effect; D = dose effect; T*S interaction; T*D interaction; S*D interaction; ns = non-significant.

Thresholds of significance used for the ANOVA analyses, according to the Dunn- Šidák correction for multiple comparisons (see Experimental procedures 2.8 and Table A2 for the corrected P values).

Significant *post hoc* differences are shown as: * = vs. vehicle, ^ = vs. BALB/c, † = vs. time interval 1. Thresholds of significance used for *post hoc* comparisons can also be found in Table A2.

**Table A8: Overview of CORT levels before (basal) and after behavioural testing (non-basal) in the ORT after diazepam or MPEP treatment**

| | ANOVA effects @ | BALB/c | | 129P3 | |
|---|---|---|---|---|---|
| | | basal | non-basal | basal | non-basal |
| vehicle | B, S | 136.4 ± 16.6 | 402.6 ± 136.7 * | 270.9 ± 45.3 | 1059.3 ± 159.8 # * |
| 1 mg/kg diazepam | | 172.3 ± 20.7 | 438.9 ± 82.7 | 325.4 ± 86.1 | 774.2 ± 164.1 |
| 10 mg/kg MPEP | | 153.0 ± 14.3 | 389.5 ± 69.9 | 382.3 ± 109.8 | 108.9 ± 47.3 |

Data are presented as mean nmol/l ± SEM. Multivariate repeated measures ANOVA was performed with basal/non-basal as within factor and strain and treatment as between factor.

@ Significant ANOVA effects: B = basal/non-basal effect; S = strain effect. Threshold of significance used for the ANOVA analyses, according to the Dunn-Šidák correction for multiple comparisons (see Experimental procedures 2.8 and Table A2 for the corrected P values)

Significant *post hoc* effects are shown as: * = vs. vehicle, # = vs. BALB/c. Thresholds of significance used for *post hoc* comparisons can also be found in Table A2.
